# Physicochemical Characteristics and In Vitro Toxicity/Anti-SARS-CoV-2 Activity of Favipiravir Solid Lipid Nanoparticles (SLNs)

**DOI:** 10.3390/ph14101059

**Published:** 2021-10-19

**Authors:** Alaa S. Tulbah, Wing-Hin Lee

**Affiliations:** 1 Pharmaceutics Department, College of Pharmacy, Umm Al Qura University, Makkah 24243, Saudi Arabia; 2 Faculty of Pharmacy and Health Sciences, Royal College of Medicine Perak, Universiti Kuala Lumpur (UniKL RCMP), Perak 30450, Malaysia; whlee@unikl.edu.my

**Keywords:** favipiravir, solid lipid nanoparticle, COVID-19, inhalation, nebulizer, coronavirus

## Abstract

The rise of coronavirus (COVID-19) cases worldwide has driven the need to discover and develop novel therapeutics with superior efficacy to treat this disease. This study aims to develop an innovative aerosolized nano-formulation of favipiravir (FPV) as an anti-viral agent against coronavirus infection. The local delivery of FPV nanoparticles (NPs) via nebulization ensures that the drug can reach the site of infection, the lungs. Solid lipid NPs of favipiravir (FPV-SLNs) were formulated utilizing the hot-evaporation method. The physicochemical formulation properties were evaluated using dynamic light scattering (DLS), Fourier-transform infrared spectroscopy (FTIR), and differential scanning calorimetry (DSC). The aerosol formulation performance was evaluated using an Andersen Cascade Impactor (ACI) at a flow rate of 15 L/min. The FPV-SLN formulation’s in vitro anti-viral activity against severe acute respiratory syndrome coronavirus 2 (SARS-CoV-2) was also evaluated using the SARS-CoV-2 pathogen (hCoV-19/Egypt/NRC-3/2020 isolate). The FPV-SLNs’ morphology was defined utilizing transmission electron microscopy, showing an irregular shape. By means of FPV-SLNs’ nebulization, a fine particle fraction of 60.2 ± 1.7% was produced with 60.2 ± 1.7%, and this finding suggests that FPV-SLNs were appropriate for inhalation drug delivery with a particle size of 537.6 ± 55.72 nm. Importantly, the FPV-SLNs showed anti-viral activity against SARS-CoV-2 with CC_50_ and IC50 values of 449.6 and 29.9 µg/mL, respectively. This study suggests that inhaled solid lipid NPs of favipiravir could potentially be used against coronavirus.

## 1. Introduction

The World Health Organization (WHO) recently listed COVID-19 as a pandemic, coupled with rising cases and global mortality rates [1,2,3]. COVID-19 is characterized by various complications ranging from moderate, self-limiting respiratory tract illness to severe pneumonia, organ failure, and death [4]. In addition, cough, fever, dyspnea, sputum production, fatigue, diarrhea, and difficulty breathing are common symptoms of this disease [5]. Current conventional therapies such as antiviral drugs appear to be effective in many cases, demonstrating that it is vital to suppress and control the replication of this virus [6,7,8]. In addition, vaccines seem to work very well against COVID-19 [9,10,11]. However, there are not yet effective anti-SARS-CoV-2 treatments. Therefore, many countries are focused on developing a therapeutic formulation with superior efficacy to combat COVID-19 infection. This can be done by repurposing existing anti-viral drugs into an inhalable formulation to deliver drugs into the lungs locally.

As the route of SARS-CoV-2 infection and subsequent disease progression is within the lungs, the direct drug inhalation into the lungs seems the most suitable route to achieve high local deposition and rapid infection alleviation. Inhaled drug delivery for coronavirus treatment is preferable [12,13] over the systemic route [14,15] to avoid systemic side effects and first-pass metabolism [16,17,18,19]. In 2020, The International Society for Aerosols in Medicine (ISAM) issued an urgent appeal to policymakers and governmental agencies to urgently develop inhaled therapies for COVID-19 [20]. The inhaled therapy approach for viral infection is not something new, considering the many examples of FDA-approved medications such as inhaled zanamivir and inhaled ribavirin to treat influenza and respiratory syncytial virus infections, respectively [20]. Nebulizers are the most widely used devices, as they can deliver a drug in a solution or suspension form with high drug concentration to the target site location. This also enhances the local therapeutic index simulation [21,22] and does not require hand/breath coordination [23].

Favipiravir (FPV) acts as a competitive RNA-dependent RNA polymerase inhibitor [24]. FPV is a purine nucleoside analog and is used as an anti-viral drug against influenza types A and B and viral hemorrhagic fever [25,26,27]. FPV has been approved for pandemic influenza strains in Japan due to its well-characterized safety profile [28]. The basis of FPV efficacy as a COVID-19 treatment is believed to be linked to the fact that FPV can inhibit viral replication and transcription [25]. Jin et al. also demonstrated that FPV was incorporated into the viral RNA strand and prevented further extensions [29]. Furthermore, Oestereich et al. found that FPV had a potential effect on the Ebola virus in mice [30]. Another in vitro study showed that FPV, as a prodrug, had an inhibitory effect against severe acute respiratory syndrome coronavirus 2 infection (SARS-CoV-2) with 61.88 μmol·L^−1^ of half-maximal effective concentration, >400 μmol·L^−1^ of half-maximal cytotoxic concentration, and >6.46 of selectivity index [26].

However, FPV is a hydrophobic molecule with low solubility in water (8.7 mg/mL) as well as a short half-life, leading to rapid renal clearance in its hydroxylated form [31]. Therefore, the use of a nanocarrier such as lipid-based nano-formulations, nano-emulsion, and polymeric nanoparticles (NPs) is a rational approach to overcome the abovementioned limitations of FPV [32,33,34]. In addition, the use of biodegradable polymers as nanocarriers has been recognized by the FDA to enhance the drugs’ stability and solubility [35,36,37,38]. Chun et al. demonstrated that the co-encapsulation of FPV and mir-323a into amphiphilic copolymersomes enhanced in vitro cellular uptake and consequently resulted in a synergistic effect in controlling H1NI virus infection [34]. In another study, FPV-loaded nano-emulsion was postulated to facilitate the binding to the virus RNA-dependent RNA polymerase, prevent the SARS-CoV-2 virus replication, and destroy the viral structure [33]. In addition, liposomes, polymeric NPs and micelles have been used in pulmonary inhalation routes to improve medication efficacy and target drug delivery [39,40,41,42,43,44,45].

Therefore, this study aims to develop a nebulized favipiravir solid lipid nanoparticle formulation (FPV-SLN) for COVID-19 treatment. This is a proof-of-concept study on the feasibility of FPV in the form of nanoparticles as an inhaled formulation to control SARS-CoV-2 infection. This formulation was evaluated in terms of its physicochemical characteristics and in vitro aerosol deposition behavior. Additionally, the FPV-SLN formulation’s efficacy and anti-viral activity were assessed with regard to the SARS-CoV-2 Egyptian strain.

## 2. Results and Discussion

This research is designed to assess the synthesis, physicochemical properties (i.e., in vitro drug release profile, aerosol performance), and suitability for lung delivery via nebulization of favipiravir solid lipid nanoparticles (FPV-SLNs). In addition, the nano-formulation was investigated for its efficacy and impact in vitro against severe acute respiratory syndrome coronavirus 2 (SARS-CoV-2) (hCoV-19/Egypt/NRC-03/2020 (Accession Number on GSAID: EPI_ISL_430820). The in vitro data confirm the model that the FPV-SLN formulation showed anti-viral activity against SARS-CoV-2, with CC_50_ and IC50 values of 449.6 and 29.9 µg/mL, respectively.

### 2.1. FPV-SLN Formulation Physical-Chemical Characterization

#### 2.1.1. Characterization of FPV-SLNs

**Figure 1:**
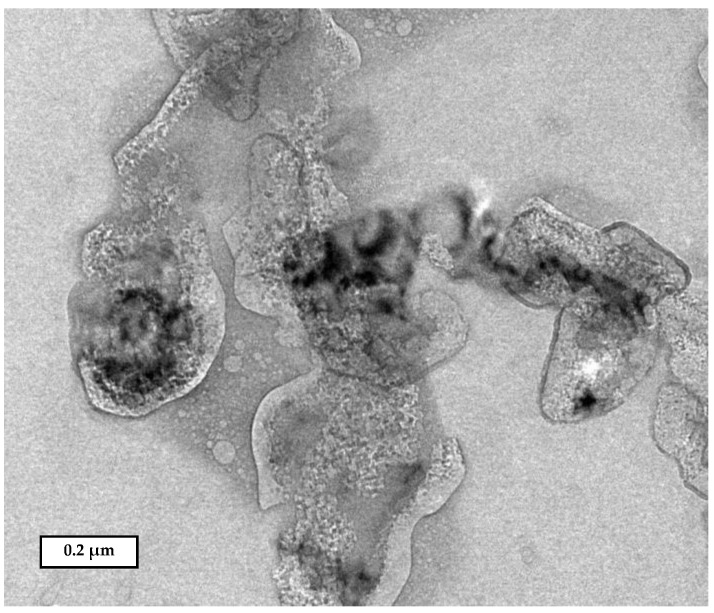
Transmission electron micrograph of FPV-SLN formulation.

This formulation showed a high FPV encapsulation efficiency inside the lipid carriers at 92.91 ± 0.06%. The lipid carrier shell that encapsulates favipiravir not only protects the drug from degradation, but also controls drug release from the NP for an extended period of time and places the drug in direct contact with the cells. Several studies have shown that encapsulation of a drug within lipid carriers/PLGA material not only improves the drug’s stability, but also increases its efficacy [39,40,46,47]. Additionally, Table 1 shows the geometric particle size distributions for FPV-SLNs, blank SLNs, and raw FPV, which were measured using dynamic light scattering (DLS). The mean diameter, polydispersity index (PDI), and surface charge of FPV-SLNs were 693.1 ± 40.3 nm, 0.655 ± 0.020, and −13.3 ± 0.3 mV, respectively. Inhaled particles smaller than 5000 nm in diameter are highly preferable for lung drug delivery [48]. The net negative charges of the NPs could be owing to the ionization of carboxylic groups in glyceryl behenate (a fatty acid in compritol 888) [49,50]. An in vivo study by Patel et al. demonstrated that negatively charged particles were more localized in rats’ lymphatics than positively and neutrally charged ones [51]. This evidence suggests that the FPV-SLN formulation could be localized in the lymphatic system to achieve higher treatment efficacy and recovery [52]. SARS-CoV-2’s pathogenesis in causing pneumonia has two phases. The early stage is marked by viral replication, which causes direct virus-mediated tissue damage. The advanced stage is marked by the infected host cells eliciting an immune response, including the recruitment of T lymphocytes, neutrophils, and monocytes [53]. Lungs and lymphatic organ injuries are linked to fatal systematic respiratory and immune malfunction in critically ill COVID-19 patients [52]. Figure 1 indicates the transmission electron micrograph (TEM) of FPV-SLNs of approximately 600 nm in size and irregular shape. These data were in alignment with the particle size distribution data measured using DLS.

#### 2.1.2. FPV-SLNs’ Drug Release Profile

**Figure 2:**
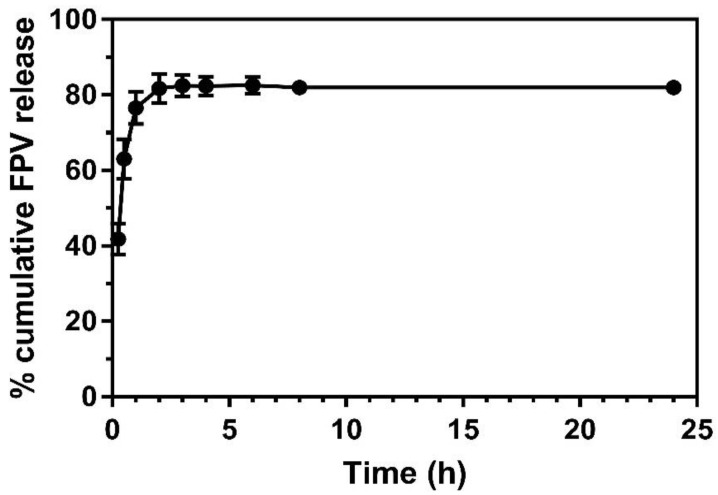
In vitro drug release across a dialysis membrane of FPV-SLNs (*n* = 3, mean ± SD).

Figure 2 demonstrates the FPV-SLN formulation’s release profiles across a dialysis membrane in experimental conditions to represent the physiological condition in vivo. Over the 24 h of the experiment, the FPV-SLN solution formulation confirmed FPV diffusion from the dialysis bag with 83% and 82% of the drug released at the 6th and 8th h, respectively. Meanwhile, approximately 77% of the drug was released after the first hour of the experiment thus indicating no retardation of drug diffusion across the dialysis membrane. It is obvious that the FPV released from SLNs was nearly complete (82 ± 0.017%) after 24 h. In an in vivo study by Videira et al., which researched the distribution of nebulized radiolabeled SLNs in rats’ lungs [53], a high number of radiolabeled particles were found in the sacrificed animals’ lungs, as well as high lymphatic uptake and lymph node distribution compared to radiolabeled lipophilic 99 m Tc-HMPAO as the control group [53]. These data recommend that the delivery of FPV via a solid lipid carrier may maintain favipiravir nanoparticles in the lung lymphatic system to treat COVID-19 patients.

**Figure 3:**
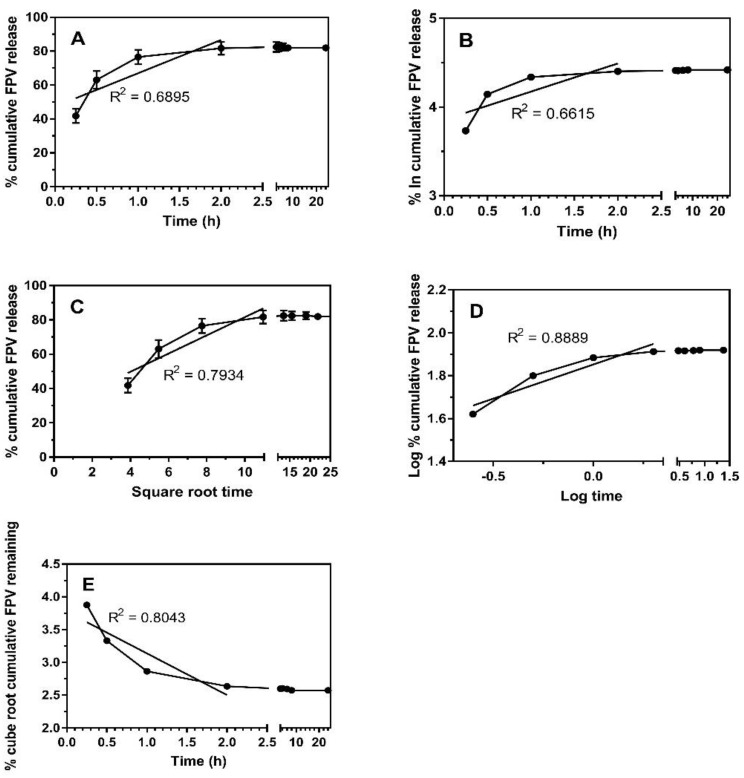
Drug release kinetics of FPV-SLNs fitted to mathematical kinetic model. (**A**) Zero order, (**B**) first order, (**C**) Higuchi model (**D**) Korsmeyer–Peppas model and (**E**) Hixson–Crowell model. It should be noted that only cumulative release of FPV until 2 h is fitted to the model. Solid black circle refers to FPV release.

The in vitro FPV release data were fitted to five different mathematical models and interpreted based on the correlation coefficient (R^2^) as shown in Table 2 and Figure 3. In general, the highest R^2^ obtained demonstrated that the drug release pattern followed the model. The mathematical models of the release kinetic profiles were fitted to release data obtained up to 2 h. The remaining FPV released after 2 h is considered saturated or equal to 100% drug release. Table 2 and Figure 3 show FPV drug release and a higher degree of the correlation coefficient for the Hixson–Crowell and Korsmeyer–Peppas models. This indicates that the change in surface area and particle size of NPs during the process of dissolution/erosion had a significant effect on drug release. A good R^2^ obtained for Korsmeyer–Peppas showed that other release mechanisms such as diffusion also occurred simultaneously in addition to dissolution/erosion of NPs.

#### 2.1.3. Fourier Transform Infrared Spectroscopy (FTIR)

**Figure 4:**
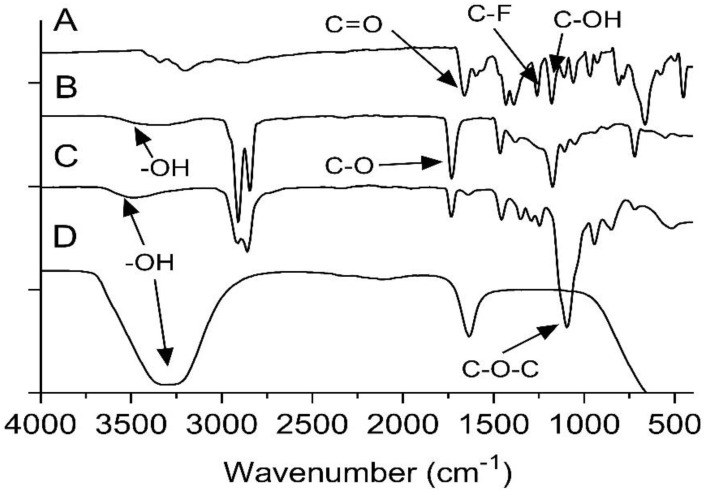
FTIR spectra of (**A**) unprocessed FPV, (**B**) Compritol 888, (**C**) Tween 80, and (**D**) FPV-SLNs.

The FTIR assessment was used to explore any peak shifts or changes in the FPV-SLN formulation when compounds such as Compritol 888 and Tween 80 were used in the preparation of nanoparticles. Figure 4A presents the FTIR of unprocessed FPV measured from 400 to 4000 cm^−1^. The stretching peaks at 1659.17, 1259.51 cm, and 1178.64 cm^−1^ were characteristic of the C=O, C-F, and C-OH stretching, respectively. The stretching of N-H groups was observed at 3340.52 and 3203.11 cm^−1^ [54]. For the Compritol 888 spectrum, the broad band observed between 3600 and 3100 cm^−1^ was assigned to the -OH stretching. Meanwhile, the peak at 1729.71 cm^−1^ indicated the presence of C-O (Figure 4B). Between 700 and 1500 cm^−1^, many bands appeared in the FTIR spectra related to the methylene groups [55]. The Tween 80 showed absorption bands related to -CH_2_, -CH_3_, and C-O-C at 2913, 2857, and 1093.81 cm^−1^, respectively. In addition, there is a stretching band at 1731.47 cm^−1^, which is due to the C=O ester group, and a strong band at 3486.33 cm^−1^ associated with the O-H (hydroxyl group) (Figure 4C) [56,57]. The FPV-SLNs’ FTIR findings show whether there was any chemical interaction between the components and the nanoparticle drug. The absorption bands at 3317.09 and 1633.62 cm^−1^ show similar spectra in bands stretching of mixed chemicals used in the preparation, thus demonstrating compatibility in the formulation.

#### 2.1.4. Thermal Properties Analysis

**Figure 5:**
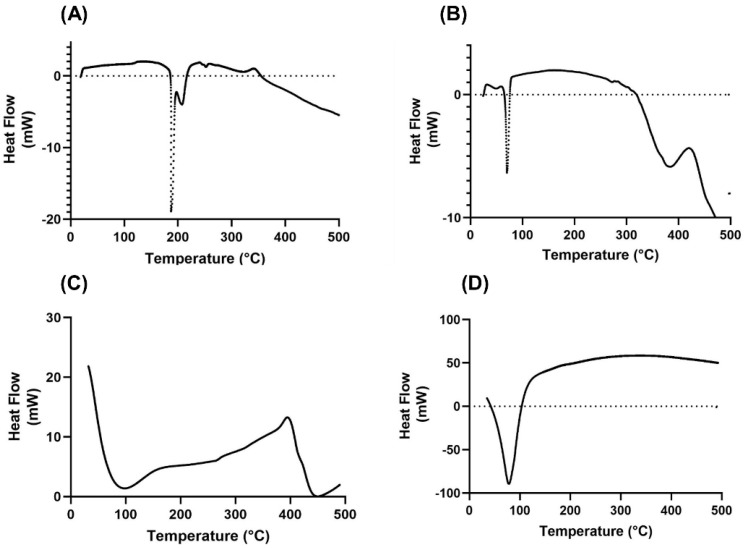
Differential scanning calorimetry thermographs of (**A**) unprocessed FPV, (**B**) Compritol 888, (**C**) Tween 80, and (**D**) FPV-SLNs.

The thermal behaviors of the FPV-SLNs and the respective raw materials (i.e., of unprocessed FPV, Glycerol behenate, FPV-SLNs, and Tween 80) are shown in Figure 5. The unprocessed FPV spotted a sharp endothermic peak at 187.06 °C, which agrees with a published research paper by Goloveshkin et al. [58]. The lipid “glycerol behenate” assessment indicated a sharp endothermic peak at 70.13 °C [59], confirming the stable β form [55], which also corresponds to their melting point and indicates crystalline behavior. However, for FPV-SLNs, a sharp endothermic peak was not observed. A broad endothermic peak shift to 78.62 °C was noted for FPV-SLNs, thus indicating FPV miscibility as a solid nanoparticle solution, which might be recognized as the glycerol behenate melting point. A similar observation was observed, in which the endothermic peak of a drug-loaded solid lipid nanoparticle using Compritol 888 and polyethylene glycol elements was shifted to 86.93 °C, representing the drug integration into the lipid matrix [60]. Additionally, the non-appearance of an endothermic peak for FPV within the FPV-SLN preparation’s thermogram indicates that it was entirely mixable inside the preparation.

#### 2.1.5. In Vitro Aerodynamic Evaluation of Nebulized FPV-SLN Formulation

**Figure 6:**
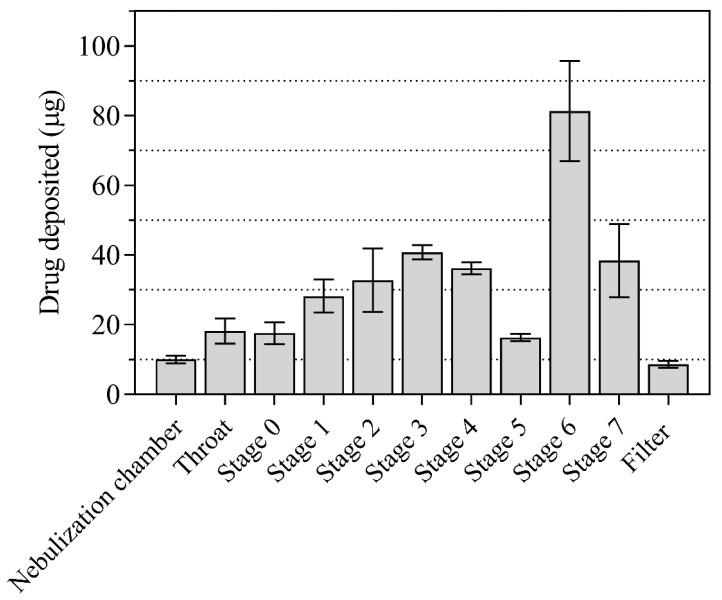
Summarized data showing ACI different stages deposition of nebulized FPV-SLN formulations (*n* = 3, mean ± SD).

The FPV-SLNs are designed to be delivered via nebulization which mimicked the nano-in-microparticles approach. The FPV-SLNs were dispersed in aqueous conditions before nebulization; consequently, each aerosolized droplet contained many FPV-SLNs. The characteristics of the in vitro aerosol deposition behavior of the nebulized FPV-SLN formulation using Andersen MKII cascade impaction (ACI) are displayed in Figure 6 and Table 3, respectively. Figure 6 shows FPV-SLNs’ deposition after nebulization on ACI stages. The data are characterized as micrograms of the total drug accumulated in the nebulization chamber, throat, filter, and each of the ACI stages over the emitted dose from the device. Aerosol performance parameters such as fine particle fraction (FPF), mass median aerodynamic diameter (MMAD), and geometric standard deviation (GSD) were calculated from the log regression plot of these data and presented in Table 3. The FPF percent is defined as the drug mass percentage placed from stage three to filter (corresponding to the cut-off size ≤5 μm) as a function of the ex-valve dose (ED). The calculated delivered dose and FPF were 332.3 ± 25.6 µg and 60.2 ± 1.7%, respectively. This suggests positive lung delivery of the FPV-SLNs and consolidates this finding for future clinical bioequivalence tests.

### 2.2. In Vitro Bio-Characterization

#### Cytotoxicity and FPV-SLNs Anti-Viral Activity

**Figure 7:**
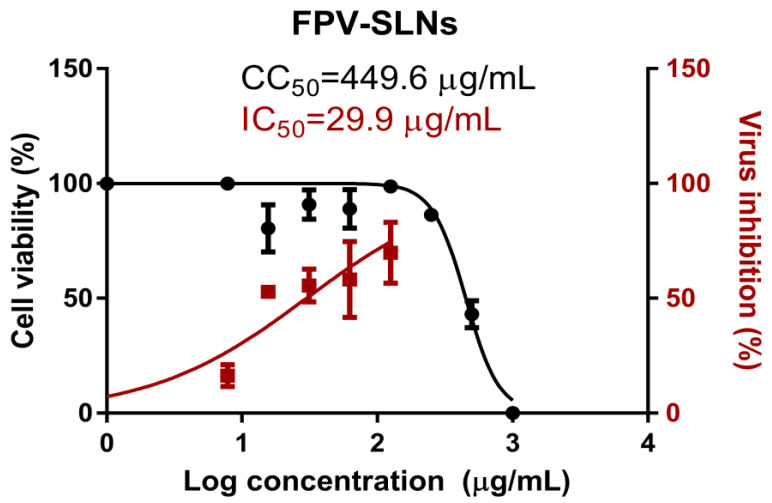
Determination of the antiviral activity of FPV-SLNs against severe acute respiratory syndrome coronavirus 2 (SARS-CoV-2). The dots represent the cell viability readouts at different treatment concentrations, and the squares represent the virus inhibition percentages of the treatment on Vero E6 cells. Data represent mean ± SD (*n* = 3).

The half-maximal cytotoxic concentration (CC_50_) was assessed to define the appropriate FPV-SLN concentrations as anti-viral activity by using the 3-(4, 5-dimethylthiazol -2-yl)-2, 5-diphenyltetrazolium bromide (MTT) assay for each experimental condition. The half-maximal inhibitory concentration (IC_50_) values were determined using the GraphPad Prism software (version 5.01, San Diego, CA, USA) via non-linear regression analysis, drawing the normalized response (variable slope) versus log inhibitor based on the outcomes of the MTT assay. Figure 7 reveals the promising in vitro antiviral properties of FPV-SLN against NRC-03-nhCoV. It was found that FPV-SLNs showed in vitro anti-viral activity against SARS-CoV-2 (Vero-E6 infected cells) with CC_50_ and IC50 values of 449.6 and 29.9 µg/mL, respectively. Selectivity index measurement was difficult due to the cytotoxicity. These results prove that the FPV fabrication using a lipid carrier showed superior anti-viral activity against NRC-03-nhCoV cells. This study demonstrated novel outcomes in which favipiravir could be repurposed into an inhalable nano-formulation as an effective treatment strategy against COVID-19 infection.

## 3. Materials and Methods

### 3.1. Materials

Favipiravir and Compritol 888 (glyceryl behenate) were acquired from Sigma Aldrich, St. Louis, MO, USA. Tween 80, chloroform, and methanol were purchased from Thermo-Fisher Inc., Pittsburgh, PA, USA. Methanol and acetonitrile were purified using a Milli-Q Plus system (Millipore Corp., Bedford, MA, USA).

The Vero-E6 cells were bought from the American Type Culture Collection, Manassas, VA, USA. Cell culture materials, including Dulbecco’s modified Eagle’s medium (DMED medium), streptomycin, penicillin, and foetal bovine serum (FBS), were purchased from Invitrogen, Waltham, MA, USA.

### 3.2. Formulation of the Favipiravir Solid Lipid Nanoparticles Formulation (FPV-SLNs)

The SLNs were formulated by applying the hot-evaporation technique as explained previously by Landh et al., including minor modifications [61]. In brief, Compritol 888 (200 mg) and favipiravir (20 mg) were suspended in 10 mL of chloroform and 5 mL of methanol, respectively. The organic solvents were evaporated at 72 °C and 150 rpm by a rotary evaporator (Heidolph, Germany). The resultant lipid–drug film included 10 mL of Tween 80 as a hot surfactant (1.5% *w*/*v*) and homogenized (HG-15D, DAIHAN Scientific Co., Ltd., Wonju. Korea) at 70 °C and 15,000 rpm for 30 min. The suspension was subsequently chilled on ice with continuous stirring until it reached 20 °C. Blank SLNs were prepared similarly without the addition of FPV.

### 3.3. Particle Characterization of FPV-SLN Formulations

#### 3.3.1. Zeta Potential, Particle Size, and Polydispersity Index Analysis

The zeta potential, particle size, and polydispersity index (PDI) of FPV-SLNs determined by dynamic light scattering (DLS) (Zetasizer Nano ZN, Malvern Analytical Ltd., Malvern, UK). Samples were diluted in ultra-purified water and investigated at 25 °C.

#### 3.3.2. Measurement of Encapsulation Efficiency

The encapsulation efficiency of freshly made FPV-SLNs was measured using the ultrafiltration method. Briefly, FPV-SLNs were placed into the ultrafiltration tubes (Amicon, Ultra-0.5, St. Louis, MO, USA) and subjected to centrifugation at 5000× *g* for 20 min. The purified solution (free FPV) was collected at the bottom of ultrafiltration tubes, and the FPV content was determined using high-performance liquid chromatography (HPLC). Encapsulation efficiency was estimated indirectly by deducting the free FPV from the overall quantity added using the following equation:

$$\mathrm{Encapsulation}\;\mathrm{efficiency}\;\left(\%\right)=\frac{\mathrm{Unfiltered}\;\mathrm{FPV}-\mathrm{filtered}\;\mathrm{solution}\;\left(\mathrm{free}\;\mathrm{FPV}\right)}{\mathrm{Unfiltered}\;\mathrm{FPV}}\times 100$$

#### 3.3.3. Transmission Electron Microscopy (TEM)

The morphologies of FPV-SLNs were evaluated utilizing transmission electron microscopy (JEM-2100 Plus Electron Microscope, JEOL, Tokyo, Japan) with an acceleration voltage of 200 kV. TEM samples were planned by drop-casting FPV-SLN suspensions on a carbon-coated copper grid and air-dried before observations.

#### 3.3.4. In Vitro FPV Release from the Nanoparticles

In vitro release analysis was applied in 50 mM phosphate buffer saline (PBS) at pH 7.4 with the dialysis bag diffusion method using a dialysis membrane with a cutoff size of 12,000–14,000 Da (Cellu. Sep, HiMedia, Mumbai, India). The dialysis bag filled with 1 mL of the sample was sealed on both sides and dialyzed against 25 mL of PBS under continuous stirring (100 rpm) at a thermostatically controlled temperature (37 ± 0.5 °C). At each predetermined time interval until 24 h, 1 mL of the sample was accumulated, and the volume of the release media was replenished by the addition of fresh PBS (1 mL) to maintain the sink condition. The cumulative percentage release (CPR) was analyzed using HPLC.

The release data were fitted to five kinetic models (zero-order, first-order, Higuchi, Korsmeyer–Peppas, and Hixson–Crowell) to find the release kinetics of both FPV from solid lipid nanoparticles.

A zero-order kinetic model is presented by Equation (1):

Q_t_ = Q_0_ + K_0_ t (1)

A first-order kinetic model is represented by Equation (2):

ln Q_t_ = lnQ_0_ + K_0_ t (2)

Meanwhile, the Higuchi, Korsmeyer–Peppas, and Hixson–Crowell models are described in Equations (3)–(5), respectively.

Q_t_ = K_H_ × t^1/2^ (3)

Q_t_/Q_0_ = K × t^n^ (4)

Q_0_^1/3^ − Q_t_^1/3^ = K × t (5)

#### 3.3.5. Fourier Transform Infrared Spectroscopy (FTIR)

FTIR was performed to confirm favipiravir’s presence inside the SLNs. About 2–3 mg of each sample (unprocessed FPV, FPV-SLNs, Compritol 888, and Tween 80) was combined with 100 mg of potassium bromide (KBr) and ground to uniform particle size and pressed into a pellet using a hydraulic press to be analyzed using FTIR (FTIR-8400s, Shimadzu, Kyoto, Japan). The detection was conducted at a wavenumber of 4000–500 cm^−1^ with 4 cm^−1^ resolutions.

#### 3.3.6. Thermal Properties Analysis

The FPV-SLNs thermal state was assessed utilizing differential scanning calorimetry (DSC) (DSC-60A, Shimadzu, Japan). About 5 mg of each sample (unprocessed FPV, FPV-SLNs, Compritol 888, and Tween 80) was sealed in an aluminum pan, and the heat flow was recorded from 25 to 500 °C under N_2_ gas flow (10 mL/min). The heating rate was set at 10 °C/min, and data were normalized for initial mass. Analysis of the samples was performed using TA-60ws software.

#### 3.3.7. High-Performance Liquid Chromatography (HPLC)

The FPV quantification was performed by HPLC (Waters 2690 Alliance, Shimadzu, Japan) supplied with a Waters 996 photodiode array detector using a C-18 Xterra column (4.6 × 250 mm and 5 μm particle size). The mobile phase consisting of 5:95 *v*/*v* acetonitrile: water and 0.1% phosphoric acid was used. The HPLC system conditions were set as follows: UV detector wavelength of 320 nm, a flow rate of 1 mL/min, and sample injection of 10 µL. Samples were purified using a 0.22 µm syringe filter. The retention and running times of the sample were 9.5 and 13 min, respectively.

#### 3.3.8. In Vitro Aerosol Performance of Nebulized FPV-SLN Using ACI

The characterization of the nebulized formulation was performed using a cooled Andersen MKII cascade impactor (ACI, Copley Scientific, UK) coupled with a jet nebulizer T-piece (VixOne jet nebulizer, AZ, USA). Each ACI part was rinsed with a mobile phase mixture and allowed to dry [62]. Before use, the ACI and its plates in situ were left in a refrigerator for 60 min at 5 °C for cooling [63]. After 1 h, the flow rate was adjusted to 15 L/min using a vacuum pump (Brook Crompton, UK) and calibrated with an electronic digital flow meter (MKS Instruments, Andover, USA). Then, the jet nebulizer T-piece was attached immediately to the ACI induction port. Two milliliters of the FPV-SLNs aliquot (200 µg/mL) formulation was added to the jet nebulizer reservoir. Three determinations were made (*n* = 3) as recommended by the Pharmacopeia [64]. The reservoir chamber of the nebulizer, ACI plates, adaptors, and the filter were washed with 20% *v*/*v* methanol for mass recovery. HPLC was used to determine the amount of FPV in each part of the ACI. The fine particle dose (FPD), MMAD, and the FPF were assessed using Copley Inhaler Testing Data Analysis Software (Copley Scientific, Nottingham, UK) impactor data.

### 3.4. In Vitro Bio-Characterization

A mixture medium of DMEM, 10% FBS, and 1% streptomycin/penicillin (strep/pen) antibiotic was used to maintain Vero-E6 cells under incubation at 37 °C with 5% CO_2_, as described previously [65]. To generate virus stock, cells were distributed into tissue culture flasks 24 h prior to infection with hCoV-19/Egypt/NRC-3/2020 isolate at a multiplicity of infection (MOI) of 0.1 in the infection medium. This medium consisted of 1 % L-1-tosylamido-2-phenylethyl chloromethyl ketone (TPCK)-treated trypsin and a DMEM mixture of 1% strep/pen and 2% FBS. After 2 h, the virus inoculum of the infection medium was swapped with a fresh infection medium and incubated for 3 days. To eliminate small particulate cell fragments from the sample, the cell supernatant was centrifuged at 2500 rpm for 5 min and collected at each specific time point. Then, the supernatant was relocated to a new falcon tube (50 mL) and titrated using the plaque infectivity assay. The SARS-CoV-2 virus experiment was performed in a Class III Biological Safety Cabinet SEA-III BSC (Germfree, Ormond Beach, FL, USA).

#### 3.4.1. The FPV-SLNs Cytotoxicity Assay on Vero-E6 Cells

The half-maximal cytotoxic concentration (CC_50_) was assessed as described previously [65]. FPV stock solutions were mixed into DMSO (10%) in ddH_2_O and diluted more with DMEM. The MTT method was used to evaluate FPV-SLNs cytotoxic activity on Vero-E6 cells.

In 96-well plates, the cells were seeded at 100 µL/mL of 3 × 10^5^ cells density and incubated at 37 °C with 5% CO_2_. After one day, different FPV-SLN concentrations (7.81–1000 µg/mL) were treated the cells in triplicate. After one day, the cell monolayers were rinsed with sterile PBS (3 times) after the supernatant was discarded. Then, each cell well was added 20 µl of MTT solution and incubated at 37 °C for 4 h followed by medium aspiration. Next, 200 µl of acidified isopropanol (0.04 M HCl in absolute isopropanol) mixed with the formed formazan crystals was added to each well. Formazan solution absorbance was assessed at λmax 540 nanometers at a 620-nanometer wavelength using a multi-well plate reader. The equation below was used to determine the cytotoxicity percentage compared to the control cells (untreated). The cytotoxicity % curve is contrasted with the sample concentration applied to assess the concentration that showed 5% cytotoxicity (CC_50_).

$$\%\;\mathrm{cytotoxicity}=\frac{\left(\mathrm{Non}-\mathrm{treated}\;\mathrm{cells}\;\mathrm{absorbance}\;-\;\mathrm{treated}\;\mathrm{cells}\;\mathrm{absorbance}\;\right)\times 100}{\mathrm{Non}-\mathrm{treated}\;\mathrm{cells}\;\mathrm{absorbance}\;}$$

#### 3.4.2. Inhibitory Concentration 50 (IC_50_) Determination

Inhibitory concentration 50 was measured as described previously [65]. Vero-E6 cells (2.4 × 10^4^) were distributed in each well of 96-well tissue culture plates. The plates were incubated overnight at 37 °C with 5% CO_2_. The cell monolayers were then washed once with PBS and subjected to virus adsorption (hCoV-19/Egypt/NRC-03/2020 (Accession Number on GSAID: EPI_ISL_430820)) in vitro to the cells at room temperature for 1 h. Later, the cells were treated with different concentrations of FPV-SLNs (7.81–125 µg/mL) and incubated at 37 °C with 5% CO_2_ for 72 h. Then, 100 μL of 4%-paraformaldehyde was used for 20 min to fix the cells. Additionally, 0.1% of crystal violet in distilled water was added to the cells for 15 min. Next, absolute methanol (100 μL) was mixed with the crystal violet dye into each well. The measurement color and its optical density were assessed at 570 nm using an Anthos Zenyth 200rt plate reader (Anthos Labtec Instruments, Heerhugowaard, The Netherlands). The IC_50_ of the FPV-SLNs is needed to decrease the virus-induced cytopathic effect (CPE) by 50%, compared to the control virus.

### 3.5. Statistical Analysis

At least three separate determinants of each result are stated as mean ± standard deviation (S.D.). The CC_50_ and IC_50_ plot illustrate the nonlinear fit of “Normalize” and “Transform” of the gained results. The GraphPad Prism 9 software was used to calculate the data value as the “best fit value.”

## 4. Conclusions

This study demonstrated the physicochemical characteristics and in vitro toxicity/anti-SARS-CoV-2 activity of favipiravir solid lipid nanoparticles. The study has further demonstrated that the formulation could be suitably reconstituted for nebulization with an FPF of 60.2 ± 1.7%. The formulation was also assessed for its anti-viral activity against SARS-CoV-2 and found to be with CC_50_ and IC50 values of 449.6 and 29.9 µg/mL, respectively. Therefore, the aerosolized FPV-SLN formulation is a promising candidate for local delivery and coronavirus treatment in the lungs. Future studies will focus on evaluating FPV-SLN formulations in animal models to assess their toxicities, efficacies, and pharmacokinetics.

## Figures and Tables

**Table 1:** Physical characteristics of FPV-SLNs, blank SLNs, and unprocessed favipiravir.

	FPV-SLNs	Blank SLNs	Unprocessed Favipiravir
Particle Size (nm)	693.1 ± 40.3	389.3 ± 26.5	1056.4 ± 181.2
Polydispersity Index	0.655 ± 0.020	0.491 ± 0.0360	0.451 ± 0.036
Zeta Potential (mV)	−13.3 ± 0.3	−14.8 ± 0.8	−11.1 ± 0.2

Data are expressed as mean ± SD of three replicates.

**Table 2:** Kinetics parameters favipiravir from the FPV-SLN formulation fitted to mathematical kinetic model. * It should be noted that only cumulative release of FPV until 2 h is fitted to the model.

R^2^ of Sample *	Zero-Order	First-Order	Higuchi	Hixson-Crowell	Korsmeyer–Peppas
FPV-SLNs	0.6895	0.6615	0.7934	0.8043	0.8889

**Table 3:** Aerodynamic characterization of nebulized aerosol from the jet nebulizer with 400 µg delivered dose of FPV-SLN formulations (*n* = 3, mean ± SD).

Results	*n* ± SD
**Calculated Delivered Dose (µg ± SD)**	322.3 ± 25.6
**Fine Particle Dose (µg ± SD)**	193.9 ± 13.1
**Fine Particle Fraction (% ± SD)**	60.2 ± 1.7
**MMAD (µm ± SD)**	3.0 ± 0.4
**GSD**	2.33 ± 0.25

## Data Availability

Data is contained within the article.
